# Research on the Interaction of Hydrogen-Bond Acidic Polymer Sensitive Sensor Materials with Chemical Warfare Agents Simulants by Inverse Gas Chromatography

**DOI:** 10.3390/s150612884

**Published:** 2015-06-02

**Authors:** Liu Yang, Qiang Han, Shuya Cao, Feng Huang, Molin Qin, Chenghai Guo, Mingyu Ding

**Affiliations:** 1 State Key Laboratory of NBC Protection for Civilians, Beijing 102205, China; E-Mails: Csy1973@139.com (S.C.); john_hf@163.com (F.H.); qinmolin@139.com (M.Q.); guochenghaiyouxiang@126.com (C.G.); 2 Beijing Key Laboratory for Microanalytical Methods and Instrumentation, Department of Chemistry, Tsinghua University, Beijing 100084, China; E-Mail: hanq14@mails.tshinghua.edu.cn

**Keywords:** sensors, hydrogen-bonded acidic polymers, inverse gas chromatography, nerve gas simulants

## Abstract

Hydrogen-bond acidic polymers are important high affinity materials sensitive to organophosphates in the chemical warfare agent sensor detection process. Interactions between the sensor sensitive materials and chemical warfare agent simulants were studied by inverse gas chromatography. Hydrogen bonded acidic polymers, *i.e*., BSP3, were prepared for micro-packed columns to examine the interaction. DMMP (a nerve gas simulant) and 2-CEES (a blister agent simulant) were used as probes. Chemical and physical parameters such as heats of absorption and Henry constants of the polymers to DMMP and 2-CEES were determined by inverse gas chromatography. Details concerning absorption performance are also discussed in this paper.

## 1. Introduction

**Figure 1:**
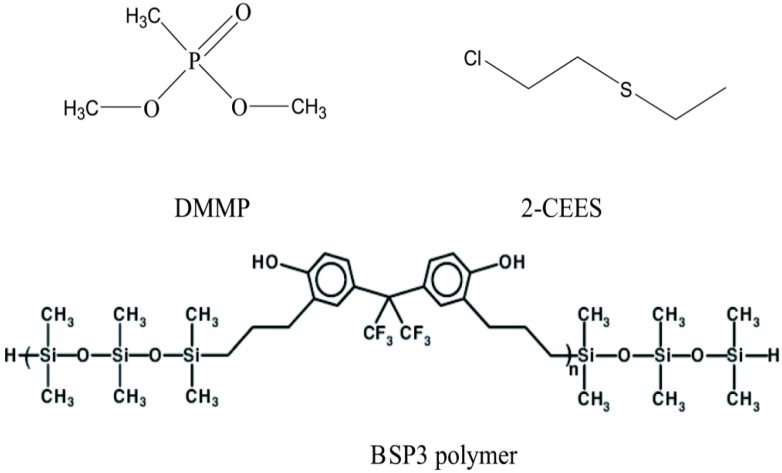
Structures of the probe molecules and hydrogen-bonded acidic polymers.

With the increase in terrorism threats in recent years, rapid on-site detection technologies for hazardous and toxic gases, including chemical warfare agents (CWAs), are drawing more and more attention. Chemical warfare agents (CWAs) are chemical substances intended for use in military operations to kill, injure or incapacitate an enemy. Given the threat posed by these threat [1,2],selective functional membranes play important roles in sensors for nerve agent gas detection. Selective materials and membranes have the ability to selectively absorb a target gas. Hydrogen-bonded acidic polymers are important materials in research on hazardous gas enrichment and detection [3,4,5,6,7,8,9].The oxygen atom of the P=O group in nerve agent gases is an electron donor, and according to the Lewis acid-base theory, nerve agent gases are hydrogen-bond based gases, so hydrogen-bonded acidic polymers should have the capability of selectively absorbing nerve agent gases. Dimethyl methylphosphonate (DMMP) is a nerve agent gas stimulant and 2-chloroethyl ethyl sulfide (2-CEES) is a blister agent simulant commonly used in research (Figure 1). Hydrogen-bonded acidic BSP3 polymers were synthesized in the laboratory and dispersed on filler particles to make stationary phases, then micro packed columns were fabricated by filling them with polymer particles and characterized by the inverse gas chromatography method [10]. Using DMMP and 2-CEES as probe molecules, some physical chemistry parameters of the sensor material such as heats of absorption and Henry’s constants were researched in this paper.

## 2. Experimental Section

### 2.1. Chemicals

All chemicals used were at least of analytical grade. Dichloromethane (Beijing Chemical Reagent Co. Ltd., Beijing, China), 102 White support silanized particle mesh number 60–80 (Beijing Chemical Reagent Co. Ltd.), 1,1,3,3,5,5-hexamethyltrisiloxane (Aldrich St Louis, MO, USA), 2,2-bis(3-allyl-4-hydroxyphenyl) hexafluoropropane (Beijing Institute of Pharmaceutical Chemistry, Beijing, China); H_2_PtCl_6_, dihydrogenhexachloroplatinate (IV) solution, Pt 20% (Alfa Chemical Reagent Co. Ltd., Tianjin, China), dimethylmethylphosphonate (DMMP) (Beijing Institute of Pharmaceutical Chemistry, Beijing, China) treated by a conventional method, and 2-chloroethyl ethyl sulfide (2-CEES, Aldrich) were used as received without further treatment. 

### 2.2. Synthesis of BSP3 Polymer

BSP3 polymer was synthesized according to Pinaki *et al*. [6,11]. 1,1,3,3,5,5-Hexamethyltrisiloxane (0.56 g, 0.0027 mol) was added to a magnetically stirred solution of 2,2-bis(3-allyl-4-hydroxyphenyl) hexafluoropropane (1.192 g, 0.0028 mol) in toluene (5 mL), using H_2_PtCl_6_ (0.07 g) as a catalyst and solution was heated to 120 °C in an oil bath. Removal of the catalyst was achieved with activated charcoal, followed by filtration. The solvent was removed using a rotary evaporator, and volatile impurities were removed by heating under vacuum for 19 h at 60 °C and then 1 h at 140 °C.

### 2.3. Fabrication of the Micro Packed Column

BSP3 polymer dissolved in dichloromethane was used to prepare polymer solutions to 1% (weight ratio), and then 102 Silanized white support was soaked in polymer solution for 24 h. Dichloromethane was removed on a rotary evaporator and dried polymer loaded supports were thus obtained. Polymer loaded support was loaded into deactivated stainless steel columns, to give polymer-loaded micro packed columns. The length of the micro packed column was 1 m.

### 2.4. Instrumentation and Procedures

Gas chromatography experiments were performed on an Agilent 7890A/5975C GC/MS (Agilent Technologies, Santa Clara, CA, USA). A flame ionization detector (FID) was used. High purity helium (99.999%, Bei Fen Co. Ltd, Beijing, China) was used as the GC used carrier gas. Liquid samples were injected by an automatic sampler; the sample injection volume was 1 μL. The injector was kept at 250 °C. The FID was kept at 250 °C. The column oven was set at a constant temperature (110 °C–160 °C) that was changed for different experiment. Flow rates of carrier gas were set at a constant rate of 2–20 mL·min^−1^, and flow rate was also changed for different experiments. The micro packed column with polymer was installed on the gas chromatograph, the column oven was adjusted to 250 °C and micro packed column was aged for 24 h. Then liquid DMMP and 2-CEES samples were injected and the corresponding chromatograms were recorded at different oven temperatures and flow rates, to investigate the retention times and retention volumes of the probes. The thermal stability of the samples was tested using a thermo gravimetric analyzer (TGA-Q5000 thermal gravimeter, TA, New Castle, DE, USA. High purity N_2_ as carrier gas). A 5-mg sample of polymer loaded support was placed in a glass tube with an inner diameter of 3-mm. Both ends of the tube were plugged with glass wool. DMMP and 2-CEES vapors were carried by 20 mL/min N_2_ gas through a saturator and then passed through the sample capillary at 50 °C. Then the support was removed from the glass tube and placed on a TGA sample plate for the TGA tests.

## 3. Results and Discussion

Standard gas chromatography is normally conducted to study a vaporized substance by conducting a separation between the mobile phase filling on the stationary phase and then detecting the components of interest in the sample. Inverse gas chromatography is studied on a stationary phase, when a non-volatile liquid sample is loaded onto the surface of a support, or a solid sample is packed into a separation column as the stationary phase. Then the interaction of a sample of the target probe molecule is studied by measuring the interaction with the stationary phase to obtain the physical and chemical parameters of the stationary phase.

To study the heat of absorption 2-CEES on BSP3 polymer, the retention time and retention volume of 2-CEES passing through the micro packed column at 110 °C–160 °C were recorded. According to the method for calculating the heats of absorption *ΔH_ads_* [12], as shown in Formula (1), we first measured the flow rate of the helium carrier gas in the column, noted the temperature of the micro packed column in the GC oven and the retention volume to make the plots of ln*Vg* (as *x*-coordinate) *vs.* 1/*T* (as *Y*-coordinate) to determine *ΔH_ads_* from the slope:

(1) $$\text{Δ}H_{ads}=-R[\vartheta (\ln V_{g}/\vartheta (1/T)]$$

where *V_g_* is the retention volume, *T* is the temperature of the micro packed column, and *ΔH_ads_* is the heat of absorption.

**Figure 2:**
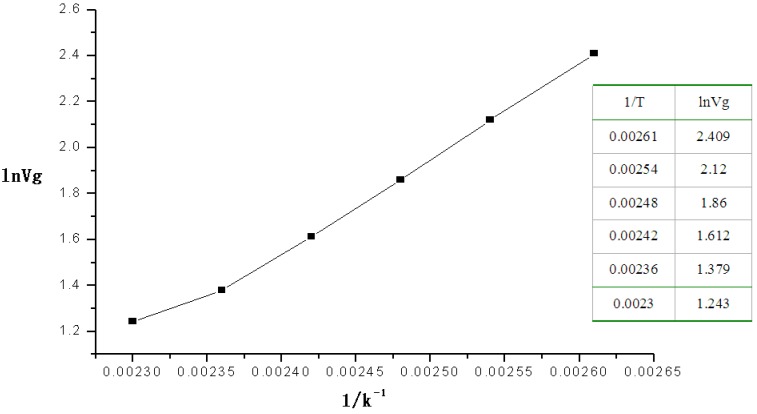
Plot of the linear relation of the heat of absorption of 2-CEES.

The retention volumes of 2-CEES passing through the micro packed column at 110°C‒160 °C were also recorded in the experiment. Figure 2 shows plots for ln*V_g_* (as the *x*-coordinate) *vs*. 1/*T* (as the *y*-coordinate).

The equation of the linear relation was *Y* = −7.7053 + 3865.04414*X*, *R* = 0.99632, from which the heats of absorption of 2-CEES on the polymer was 32.12 kJ·mol^−1^ at 110–160 °C.

**Figure 3:**
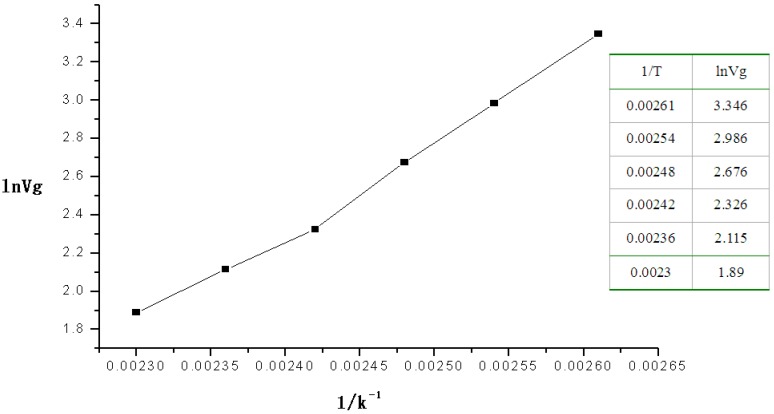
Plot of the linear relation of the heat of absorption of DMMP.

Next the retention volumes of DMMP passing through the micro packed column at 110–160 °C were recorded. Figure 3 shows the plot for ln*V_g_* (as the *x*-coordinate) *vs.* 1/*T* (as *y-*coordinate).

The equation of the linear relation was *Y* = −9.13674 + 4769.5082*X*, *R* = 0.9963, from which the heat of absorption of DMMP on the polymer at 110–160 °C was 39.65 kJ·mol^−1^.

According to the Henry constant calculation method using inverse gas chromatography, the Henry constant K’can be calculated by Formula (2):

(2) $$\text{μ}=\frac{L}{V_{r}}[\varepsilon +(1-\varepsilon )RT\rho_{c}K\prime ]$$

where the retention time is µ; the length of the column is *L*; the flow rate of the carrier gas is *V_f_*; the porosity is *ε*; *R* is the gas constantand ρ_c_ is the density; From the linear regression of µ and 1/*V_f_* in the plots according to Formula (2) the Henry constant *K*, can be calculated from the slope of the plots.

**Figure 4:**
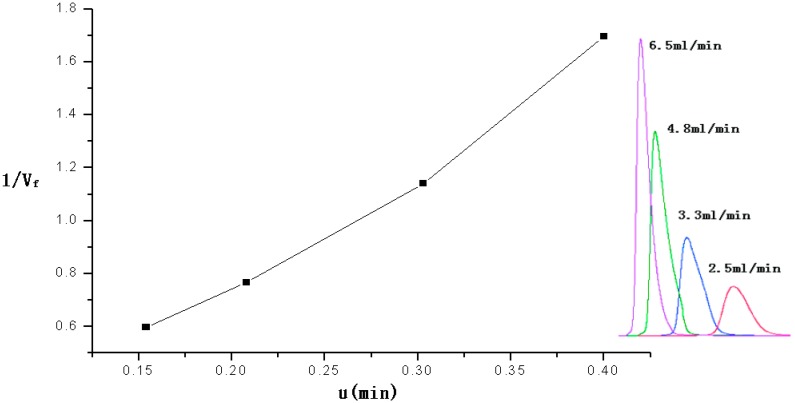
Plot of the linear relation between the retention time of 2-CEES and the reciprocal of the flow rate.

The retention time of 2-CEES were recorded when it was passed through the micro packed column at different flow rates（2.5–6.5 mL/min. The packed column was at a constant temperature of 160 °C. Figure 4 shows the plot of µ (as *x*-coordinate) *vs.* 1/*V_f_* (as *y*-coordinate).

The equation of the linear relation was *Y* = 0.00607µ + 3.81*X*, so the Henry constant of 2-CEES was 3.81 at 160°C.

The retention time of DMMP was also recorded while passing through the packed column at different flow rates (5–20 mL/min). The packed column was held at a constant temperature of 160 °C. The equation of the linear relation was *Y* = −0.01271 + 0.08794*X*, so Henry constant of DMMP was 0.0879 at 160°C.

As we can see from the heats of absorption, the heat of absorption of DMMP is larger than that of 2-CEES, The absorption value corresponds to a chemical absorption process. The large value may be related to the formation of hydrogen bonds between the hydrogen-bonded acidic polymers and the organophosphorus simulated agent. The Henry constants show the probe moleculeswere distributed between the gas phase and stationary phase. The Henry constant of DMMP was 0.0879 at 160 °C, while the Henry constant of 2-CEES was 3.81 at 160 °C. The smaller value of DMMP than 2-CEES, shows that the DMMP probe molecule is distributed in the stationary phase with priority over 2-CEES, *i.e*., the polymer had a strong absorption ability towards DMMP.

From the TGA spectra of BSP3 polymers saturated with 2-CEES, we see that 1 g of polymer loaded support can absorb 0.0124 g of DMMP, while the result for 2-CEES was 0.0096 g, confirming the strong absorption ability of the polymer for DMMP.

## 4. Conclusions

Inverse gas chromatography was used in this paper. The hydrogen-bonded acidic polymer BSP3 was dissolved in a solvent, and the obtained solution was dispersed on a support as the stationary phase in micro packed columns. Using the polymer stationary phase as absorbing carrier, and DMMP and 2-CEES as probes, the heats of absorption on BSP3 polymer were researched in this paper. The heat of absorption of 2-CEES was 32.12 kJ·mol^−1^ and that of DMMP was 39.65 kJ·mol^−1^. The Henry constant of 2-CEES was 3.81 and that DMMP was 0.0879, indicating the polymer had strong absorption ability towards DMMP.
